# Discounting of Hyper-Palatable Food and Money: Associations with Food Addiction Symptoms

**DOI:** 10.3390/nu15184008

**Published:** 2023-09-16

**Authors:** Joseph S. Bellitti, Tera L. Fazzino

**Affiliations:** 1 Department of Psychology, The University of Kansas, Fraser Hall, 1415 Jayhawk Blvd., Lawrence, KS 66045, USA; jbellitti@ku.edu; 2 Cofrin Logan Center for Addiction Research and Treatment, University of Kansas, 1000 Sunnyside Ave, Lawrence, KS 66045, USA

**Keywords:** delay discounting, choice impulsivity, food choice, addictive behaviors

## Abstract

Introduction: Delay discounting (DD), the tendency to prefer small, immediate rewards over larger, delayed rewards, is associated with health-risk behaviors. The study examined associations between DD for money and hyper-palatable foods (HPF) with food addiction (FA) symptoms among a general population sample. Methods: Participants (N = 296) completed an adjusting DD task that consisted of a single-commodity condition with HPF as the reward (HPF now vs. HPF later) and cross-commodity conditions comparing money and HPF (money now vs. HPF later; HPF now vs. money later). The Yale Food Addiction Scale 2.0 was used to assess FA symptoms. Zero-inflated negative binomial regression models tested whether discounting of HPF and money was associated with FA symptoms. Results: Findings indicated there were no significant associations between DD and FA symptoms in the single-commodity HPF condition (logit: OR = 1.02, *p*-value = 0.650; count: IRR = 1.04, *p*-value = 0.515). There were no significant associations among cross-commodity conditions comparing money now vs. HPF later (logit: OR = 0.96, *p*-value = 0.330; count: IRR = 1.02, *p*-value = 0.729) or conditions comparing HPF now vs. money later (logit: OR = 1.02, *p*-value = 0.682; count: IRR = 0.92, *p*-value = 0.128) and FA symptoms. Conclusions: Discounting HPF may not be a key behavioral feature among individuals who endorse FA symptoms.

## 1. Introduction

Delay discounting (DD) is a behavioral economic construct that has been consistently associated with risky substance use. DD characterizes the tendency to choose smaller immediate rewards over larger delayed rewards [1,2]. A robust body of research has demonstrated that elevated DD is associated with the use of a variety of substances (e.g., tobacco, alcohol, cannabis, and stimulants; for a meta-analytic review, see [3]). Most DD research has examined DD by using a single commodity in a task, most commonly money [4]. However, in an emerging area of research, DD has also been assessed via cross-commodity tasks, which provide choices between one commodity available immediately (e.g., alcohol) and a different commodity available at a delay (e.g., money). By changing the delayed outcome among multiple tasks and using clinically relevant commodities, cross-commodity DD tasks can provide more nuanced insight into patterns of choice. For example, when assessed via cross-commodity DD tasks, individuals who engaged in heavy substance use had lower DD in conditions in which their substance of choice was available at a delay (e.g., alcohol, cannabis) [5,6]. This suggested that individuals who use substances may also be willing to wait for larger amounts of their substance of choice. Taken together, DD may be a useful paradigm to conceptualize choices regarding substance use.

Given that DD has been robustly associated with substance use behavior, researchers have expanded the use of DD to understand other risky behaviors such as gambling, risky sex, and compulsive shopping [3]. However, research has largely overlooked the potential application of DD to food addiction (FA), a phenomenon that may present a risk to physical and mental health [7]. FA is assessed with criteria that parallel DSM-5 substance use disorder criteria, and the target substance of FA is considered palatable food [8]. Individuals with FA may share commonalities in symptom presentation with individuals with substance use disorder due to their parallel criteria and may also exhibit other characteristics that are commonly observed among individuals with substance use disorder, such as impulsivity. For example, a recent systematic review reported that several facets of impulsivity (e.g., disinhibition) have been consistently associated with FA among individuals who have obesity as well as community and undergraduate samples [9]. FA symptoms have been associated with greater choice impulsivity, urgency, and likelihood to compulsively consume foods among a mixed community and undergraduate sample [10]. Furthermore, a literature review suggested that individuals with FA may consume palatable foods despite long-term health consequences [11], indicating that individuals with FA may prefer the immediate reward of palatable foods in favor of long-term rewards. Thus, DD of palatable food may be an important process to examine among individuals with FA symptoms.

FA may also include elements of waiting/planning for future food intake, which holds relevance for discounting processes [12]. Specifically, as indicated by FA criteria, individuals with FA symptoms may spend substantial time seeking/consuming palatable food [12,13]. This suggests that individuals with FA symptoms may plan to consume rewarding foods in the future and thus may be willing to wait longer to receive larger amounts of palatable foods. From a DD framework, individuals with FA symptoms may be willing to invest time to obtain palatable foods in the future, even when presented with a strong immediate reward. This premise is consistent with prior cross-commodity DD studies that found that individuals with substance use disorder symptoms were willing to wait for their substance of choice, even when presented with a strong alternative commodity (e.g., money) [5,6]. Thus, examining whether individuals with FA symptoms are willing to wait for palatable foods is important to understand whether DD processes operate similarly to those observed with substance use disorders.

Only two prior studies in the literature examined the role of DD in FA symptoms. Both studies used single-commodity DD tasks with money as the reward, and the results were inconsistent. One study indicated that FA symptoms were associated with elevated DD (i.e., preference for immediate money) among a mixed community and undergraduate sample [10]. However, another study found that, among a general population sample, DD of money was not associated with greater endorsement of FA symptoms [14]. Thus far, no studies have used a commodity with specific relevance to FA (i.e., palatable foods). Furthermore, prior studies have yet to use cross-commodity DD tasks to examine the discounting of palatable foods and money, which may provide a more nuanced understanding of choices for rewarding commodities among individuals with FA symptoms [15].

The purpose of the current study was to examine the DD of hyper-palatable food (HPF) and money and its association with FA symptoms. We hypothesized that among single and cross-commodity DD tasks, a greater willingness to wait for HPF when available at a delay and a relative preference for HPF when available immediately would be associated with endorsement of higher FA symptoms.

## 2. Materials and Methods

### 2.1. Study Procedure

The Institutional Review Board at the University of Kansas approved the study. This study was conducted using Amazon Mechanical Turk (MTurk), an online platform used to collect survey data for research. Participants provided informed consent before completing a DD task and surveys regarding eating behavior. Study responses remained anonymous, and the study took approximately one hour to complete. Participants were compensated USD 4.50.

### 2.2. Participant Recruitment

Study surveys were released in six batches on MTurk to facilitate the recruitment of participants with varying schedule availability. Eligibility criteria were as follows: (1) Age 18–65; (2) current US residency; (3) MTurk quality approval rating of ≥99%; (4) completed ≥1000 studies on MTurk. Eligibility criteria were selected in accordance with MTurk data quality assurances [16] and to align with study measures that used US foods.

### 2.3. Measures

#### 2.3.1. Delay Discounting Task

The computer-based discounting task consisted of both single and cross-commodity conditions. Money (US dollars) and HPF commodities were used in the discounting conditions. HPF items were identified using the standardized definition of HPF [17] and from a list of 12 options, participants selected their preferred HPF (see [18] for details) for the DD task. Participants were given a choice of HPF to ensure the task would align with individual food preferences. The single-commodity task consisted of two conditions: (1) money now vs. money later; (2) HPF now vs. HPF later. The cross-commodity conditions included the following: (1) money now vs. HPF later; (2) HPF now vs. money later (Table 1). In total, participants completed eight discounting conditions and 240 trials.

An adjusting amount task was used to measure DD. Adjusting amount tasks are efficient and reliable measures of DD that provide more fine-grained detail regarding choice patterns in comparison to non-adjusting measures (e.g., the monetary choice questionnaire) [19]. For each trial in the task, participants indicated their preferred choice for either a smaller reward available immediately (e.g., USD 5) or a larger reward available at a delay (e.g., USD 25 in one week). Depending on the participants’ selection of reward in each trial, the next immediate reward was adjusted. If the immediate reward was selected, the immediate reward presented in the subsequent trial was adjusted down by 50% of the prior adjustment [19]. If the delayed reward was selected, the immediate reward presented in the subsequent trial was adjusted up by 50% of the prior adjustment [19]. Responses over six trials were used to determine each participant’s indifference point. This process was repeated over five delay periods (1 day, 1 week, 1 month, 6 months, and 1 year) within each condition. The indifference point at each delay period was used to calculate the k value to represent the DD rate used in analyses [19].

Participants completed each commodity condition twice, once for commodity amounts that were smaller in magnitude: That is USD 10 or 4 servings of food (USD 2.50 per serving × 4 servings = USD 10 equivalent in food) and a second time for larger magnitude amounts: USD 100 or 40 servings of food (USD 2.50 per serving × 40 servings = USD 100 equivalent in food). The initial values for each immediate reward were calculated as 50% of the delayed reward (see Table 1). For example, the initial choice for the single-commodity HPF condition in the small magnitude condition was two servings of HPF now, which represented 50% of the delayed choice (4 servings of HPF). To compare discounting rates across money and food commodities, an exchange rate was set at USD 2.50 equals one serving of food (based on US serving size). The monetary value aligned with the general market value of one serving of each food used in this study.

#### 2.3.2. Yale Food Addiction Scale 2.0 (YFAS)

The YFAS is designed to assess symptoms of FA in accordance with DSM-5 substance use disorder criteria [20]. The YFAS assessed 11 symptoms of FA over the past month via 35 items. Items are scored via a 7-point Likert scale ranging from 0–7 (never-every day). Each of the 11 FA symptoms is assessed with several question items (2–5 items) on the YFAS, and a symptom criterion is considered met if at least one corresponding item meets an established threshold (e.g., once a month). Thresholds varied by item and were established in the initial validation of the questionnaire [20]. For the present study, total FA symptom count was used as an outcome to maximize statistical power and reflect FA severity along a continuum [10,14]. Higher scores reflected greater addictive eating behavior. Prior literature has indicated the YFAS has convergent validity with other measures of eating pathology (i.e., binge eating) and impulsivity [21], as well as body mass index and eating disorder severity [22]. Kuder-Richardson’s alpha reliability coefficient was calculated in accordance with recommendations from Meule & Gearhardt (2019) [23] and was excellent for the present study (α = 0.92).

#### 2.3.3. Hunger

Before completing the DD task, participants were asked to rate their current hunger level using a 100-mm visual analog scale (VAS; 0 = Not Hungry at All, 100 = Very Hungry). Prior studies have indicated that levels of hunger may influence DD [24,25] and thus hunger was included as a covariate in analyses.

### 2.4. Data Analysis Plan

#### 2.4.1. Calculation of Delay Discounting Parameter

Data analysis was conducted using R statistical software [26]. Mazur’s hyperbolic function was used to estimate each participant’s DD rate within each condition [27]. Mazur’s hyperbolic formula is V = A/1 + kD, in which V is the discounted value of a delayed outcome (i.e., indifference point), A is the commodity amount, D is the delay in days, and k represents the estimated DD rate (i.e., the delayed commodity loses subjective value over time). A natural logarithm transformation was used to address the positive skew in the distribution of k values for parametric analysis [28]. The transformed ln(k) values were used in analyses with higher ln(k) values, suggesting a tendency to choose smaller immediate rewards over larger delayed rewards relative to lower ln(k) values.

#### 2.4.2. Statistical Analyses

Pearson correlation analyses were conducted to test correlations among ln(k) values for single and cross-commodity DD conditions and the YFAS symptom score. Analyses were also conducted to establish the presence of a magnitude effect, which would serve as a methodological check to determine whether smaller magnitudes were discounted more steeply than larger magnitudes, in accordance with the DD literature [29,30,31,32]. Therefore, a two-way analysis of variance (ANOVA) assessed differences in discounting between small and large magnitudes for single-commodity conditions consisting of HPF and money.

For the main study analyses, visual graphing techniques were used to investigate model assumptions. Q-Q plots indicated substantial deviation from normality, and model assumptions for linear regression were not met. The distribution of the outcome (i.e., YFAS scores) was negatively skewed, as approximately 71% of values equaled zero, and the data had substantial overdispersion. To address both distributional characteristics, zero-inflated negative binomial models (ZINB) were used in analyses [33,34,35]. ZINB models yield a two-part model: (1) a binary logit model to predict excess or structural zeroes, and (2) a count model based on a negative binomial distribution. Results from the binary models are interpreted via an odds ratio that indicates the odds that an individual would exhibit zero symptoms of FA more so than expected by chance [36]. Results from the count model are interpreted via an incidence risk ratio that indicates the expected rate at which an individual would exhibit symptoms of FA.

A series of ZINB models were constructed to test whether a greater willingness to wait for HPF when available at a delay and a relative preference for HPF when available immediately were associated with FA symptoms. To test the association between discounting of HPF in single-commodity conditions and FA symptoms, a ZINB model was constructed with the ln(k) value from the single-commodity condition (i.e., HPF now vs. HPF later) as the predictor variable and the YFAS symptom score as the outcome. To test the association between discounting of HPF in cross-commodity conditions, two ZINB models were constructed with the ln(k) value from the money now vs. HPF later and HPF now vs. money later conditions, respectively, as the predictor variable and the YFAS symptom score as the outcome. Ln(k) values from the single-commodity money conditions were calculated and included for comparison. Hunger was included as a covariate in all analytic models. A bootstrapping technique was used via a random sample of 1200 cases to calculate bias-corrected confidence intervals for the models, consistent with recommendations in the literature for zero-inflated models in R [37,38,39].

#### 2.4.3. Data Quality Criteria and Missing Data

Criteria from Johnson and Bickel (2008) [40] were used to identify non-systematic discounting data. Because the criteria were developed and validated from single-commodity studies that used money as the reward, they were applied to single-commodity money data in the current study. Individuals who violated both Johnson & Bickel (2008) [39] criteria in the single-commodity money condition were removed prior to analysis. This process resulted in the removal of 4.1% of participants in the small magnitudes (*n* = 12) and 3.7% of participants in the large magnitudes (*n* = 11). The final sample consisted, therefore, of N = 284 in analyses using small magnitude conditions and N = 285 in analyses using large magnitude conditions.

Participant data were evaluated for quality via attention check completion. Criteria to remove participant data due to inattention were established as failing more than one attention check. Zero participants met this criterion, and therefore no participants were removed from analysis due to inattention. All individuals completed the YFAS, and there was no missing data.

## 3. Results

### 3.1. Participants

Sample characteristics of the total sample (N = 296) are provided in Table 2. Approximately half the participants identified as men (57%). The sample was predominantly White/Non-Hispanic (73%) and had some form of employment (i.e., full or part-time; 73%). Approximately 29% of the sample endorsed at least one symptom of FA. Among those who endorsed FA symptom(s), the mean YFAS symptom score was 4.29 (SD = 3.11). After removing participants due to low data quality, the final samples were N = 284 and N = 285 for small and large magnitude analyses, respectively.

### 3.2. DD Values and Magnitude Effect

The Supplementary Materials section includes figures (Figures S1 and S2) that display the median indifference points as a function of the time delay using Mazur’s hyperbolic equation to assess the line of best fit [27]. Figures were constructed for both large and small magnitude conditions, as is typical in DD literature. Pearson correlation analyses revealed small correlations among ln(k) and YFAS symptom scores across conditions for both small and large magnitude conditions (presented in Supplementary Materials, Tables S1 and S2). Results of the ANOVA model yielded a main effect for magnitude, suggesting that across money and HPF commodities, individuals demonstrated significantly greater DD of the small magnitude reward relative to the large magnitude reward (Mean ln(k) difference = 0.76; F(1, 275) = 49.50, *p* < 0.001). Pairwise comparisons indicated that individuals exhibited greater DD of the small reward relative to the large reward for money (Mean ln(k) difference = 0.91; t = 10.48, *p* < 0.001) and HPF (Mean ln(k) difference = 0.60; t = 3.07, *p* = 0.002). Thus, the presence of a magnitude effect for both money and HPF yields confidence in the interpretability of our DD data.

### 3.3. Zero-Inflated Negative Binomial Analyses

Results from the ZINB analyses for the primary aims in the small magnitude condition indicated there were no statistically significant associations between either ln(k) values from single-commodity HPF conditions (i.e., HPF now vs. HPF later) or ln(k) values from cross-commodity conditions (i.e., HPF now vs. money later and money now vs. HPF later) and FA symptom score (Table 3). Findings indicated that a willingness to wait for HPF available at a delay and a preference for HPF available immediately were not significantly associated with FA symptoms (Table 3). This pattern was observed in both single and cross-commodity conditions and across both small (Table 3) and large (presented in Supplementary Materials, Table S3) magnitude conditions. Results did reveal a single significant association in the logit portion of the money vs. money condition in the small magnitude model (OR = 0.89, *p* = 0.049). The logit model of ZINB analysis predicted excess zeroes. Thus, this finding indicated that for every one unit increase in ln(k) in single-commodity money conditions, there was an 11% reduction in the odds of having zero FA symptoms, relative to chance (e.g., not experiencing symptoms during the measured period).

## 4. Discussion

The current study examined whether specific patterns of DD commonly observed among individuals with substance use disorder symptoms may be present among individuals with FA symptoms in a general population sample of adults. The results did not support the premise that preferential selection of HPF when available immediately or at a delay was associated with greater endorsement of FA symptoms (count portion of the ZINB model) or likelihood of having zero FA symptoms relative to chance (logit portion of the ZINB model). Thus, findings indicated that DD may not be a key behavioral feature among individuals who endorse FA symptoms sampled from the general population.

Our overall results align with the findings of one of two existing studies that evaluated DD and FA symptoms. Specifically, a recent study by Minhas et al. [14] indicated that elevated DD was not significantly associated with FA symptoms among a general population sample of adults, consistent with our findings. However, another study suggested elevated rates of single-commodity DD were associated with greater FA symptoms among a general population sample [10]. In contrast to our study and Minhas et al. [14], the study conducted by Vanderbroek-Stice and colleagues [10] did not address non-systematic DD data. The inclusion of potential non-systematic DD data could explain the discrepancy between findings, as outliers in the data may influence study findings in some cases [40]. The use of standardized criteria to address non-systematic DD data improves confidence in our findings and those from Minhas et al. [14], which taken together suggest that DD may not be a salient factor among individuals with higher FA symptoms. However, given the preliminary nature of the literature, more work is needed to examine this possibility.

Our findings suggest that individuals who endorse symptoms of FA may not exhibit elevated rates of DD for HPF, the target substance of FA. Notably, DD is not a specific criterion of FA, which conceptually indicates that FA symptoms can exist without this behavioral feature. For example, symptoms of craving and tolerance may drive repeated and escalating use of HPF over time, which may lead to negative physical, psychological, or social consequences [7,11,20]. Furthermore, given that DD is a behavioral economic construct, our results should be interpreted in the context of a behavioral economic theory of addiction via the reinforcer pathology model [41]. The reinforcer pathology model suggests that repeated substance use occurs through two primary yet distinct sets of behavior: (1) excessive preference for immediate rewards (i.e., DD); (2) persistently high valuation of rewards (i.e., drug demand; 2, 41). In this model, each pattern of behavior can independently contribute to a greater risk of developing substance use disorder. Thus, it is possible that the high valuation of HPF may instead be a behavioral feature that is more robustly associated with greater FA symptom endorsement. This is a premise that should be examined in future research.

Considering the body of literature on DD more broadly and as related to substance use disorder, our findings are overall inconsistent with prior work. Prior research has indicated that individuals who use substances among the general population may preferentially select their drug of choice when available immediately [15] and may be willing to wait for their substance of choice when available at a delay [5,6]. Thus, our findings may highlight a potential behavioral difference among individuals with FA symptoms as related to DD, relative to individuals with substance use disorder symptoms. However, future research is needed to replicate these findings. Another consideration for this difference is that individuals may not generally need to wait to access HPF in the food environment given the widespread accessibility of HPF [42]. The saturation of HPF within the broader food environment is an important contextual factor that requires consideration for the investigation of key behaviors associated with FA symptoms.

### Limitations

This study had several limitations. First, the DD task used hypothetical rewards for money and HPF. Prior literature has indicated that people may discount hypothetical rewards differently than real rewards, as real rewards may decrease an individual’s willingness to take risks [43,44]. Thus, using hypothetical rewards may have reduced the ecological validity and generalizability of our study. However, recent literature has indicated that responses in DD for hypothetical monetary and food rewards align with those for real monetary and food rewards [25,45], which suggests this limitation may have been mitigated to some degree. Next, our outcome was comprised of a small proportion of individuals who endorsed symptoms of FA (29%) compared to those who did not. While ZINB models are designed to address this limitation, it is possible the variability in the distribution of FA symptom endorsement was insufficient to effectively capture the anticipated differences in DD patterns. Therefore, further research should investigate the relationship between single and cross-commodity DD and FA symptom endorsement among samples with greater dispersion. Finally, this study sample was comprised of predominantly White participants and those who had some college education. Therefore, findings may not be generalizable to individuals from minoritized racial or ethnic communities or to individuals without some college education.

## 5. Conclusions

Our overall findings suggest that delay discounting (DD) of hyper-palatable foods (HPF) may not be a notable behavioral characteristic among individuals who endorse food addiction (FA) symptoms. Findings were in contrast with the broader substance use disorder literature that has identified individuals with substance use disorder symptoms as being more likely to choose their target substance when available immediately and to wait for their preferred substance when available at a delay. However, there may also be nuances in how individuals interact with HPF when compared to other substances, given its wide availability. Nevertheless, further work is needed to identify behavioral features other than patterns of DD that may better explain processes involved in FA (e.g., high valuation of HPF).

## Figures and Tables

**Table 1:** Single and Cross-commodity Conditions used in Delay Discounting Task.

Condition Type	Condition	Magnitude of Delayed Commodity
Single-commodity	Money vs. Money	USD 10
	Money vs. Money	USD 100
	HPF vs. HPF	4 servings
	HPF vs. HPF	40 servings
Cross-commodity	Money vs. HPF	4 servings
	Money vs. HPF	40 servings
	HPF vs. Money	USD 10
	HPF vs. Money	USD 100

Note: HPF = Hyper-Palatable Food: Immediately available commodities are listed first, followed by larger delayed commodities for each condition. The exchange rate was set at USD 2.50 = 1 serving of food.

**Table 2:** Study Sample Characteristics.

Variable	Mean (SD) or N (%) (N = 296)
**Gender**	
Man	170 (57.4)
Woman	125 (42.2)
Transgender	1 (<1)
**Race/Ethnicity**	
White/Non-Hispanic	215 (72.6)
White/Hispanic	14 (4.7)
Black/Non-Hispanic	23 (7.8)
Asian/Non-Hispanic	28 (9.5)
Native American	3 (1.0)
Multiracial/Ethnicity	13 (4.4)
**Age**	38.27 (11.01)
**Education**	
<High-School GED	1 (<1)
High School GED or Equivalent	31 (10.5)
Some college, no degree	58 (19.6)
Post-secondary degree	131 (44.3)
Graduate/Professional degree	47 (15.9)
Not Reported	28 (9.5)
**Income**	
<20 k	29 (9.8)
20 k–49,999	81 (27.4)
50 k–99,999	112 (37.8)
100 k+	46 (15.5)
Not Reported	28 (9.5)
**Employment**	
Full/Part-time	216 (72.9)
Unemployed/Disabled	49 (16.6)
Not Reported	31 (10.5)

Note. Demographics data were obtained from our survey. For race/ethnicity, participants were allowed to select more than one option.

**Table 3:** Zero-Inflated Negative Binomial Regression with Small Magnitude Discounting Conditions and Food Addiction Symptoms (N = 284).

Condition	Model (Count/Logit)	IRR/OR (95% CI)	SE	*p*-Value
HPF vs. HPF	Count	1.02 (0.91–1.14)	0.05	0.650
	Logit	1.04 (0.73–2.99)	0.06	0.515
Money vs. HPF	Count	0.96 (0.89–1.06)	0.04	0.330
	Logit	1.02 (0.72–1.32)	0.05	0.729
HPF vs. Money	Count	1.02 (0.92–1.11)	0.05	0.682
	Logit	0.92 (0.44–1.04)	0.05	0.128
Money vs. Money	Count	1.06 (0.97–1.18)	0.05	0.232
	Logit	0.89 (0.20–1.10)	0.06	0.049

Note: Hunger was included as a covariate in all models. CI = Confidence Interval; HPF = Hyper-Palatable Food; IRR = Incidence Rate Ratio; OR = Odds Ratio; Logit refers to binary portion of the zero-inflated negative binomial model.

## Data Availability

The data presented in this study are available on request from the corresponding author. The data are not publicly available due to privacy and ethical restrictions.

## Supplementary Materials

The following supporting information can be downloaded at: https://www.mdpi.com/article/10.3390/nu15184008/s1, Table S1: Pearson Correlations between Single and Cross-Commodity Conditions and Large Magnitude Conditions and YFAS Symptom Score; Table S2: Zero-Inflated Negative Binomial Regression with Large Magnitude Discounting Conditions and Food Addiction Symptomology (N = 285); Figure S1: The median indifference point as a function of delay in days for small magnitude conditions using Mazur (1987) [27] hyperbolic model; Figure S2: The median indifference point as a function of delay in days for large magnitude conditions using Mazur (1987) [27] hyperbolic model.
