# Supplementary material for: Discounting of Hyper-Palatable Food and Money: Associations with Food Addiction Symptoms
Source: Nutrients. 2023 Sep 16;15(18):4008. doi: 10.3390/nu15184008 (PMC10536694; doi:10.3390/nu15184008)
Supplement: Supplementary file 1 [file nutrients-15-04008-s001.zip › nutrients-2536372-supplementary.pdf]

Table S1. Pearson Correlations between Single and Cross-commodity Conditions and Small Magnitude Conditions and YFAS Symptom Score.

Conditions	HPF vs. HPF	HPF vs. Money	Money vs. HPF	Money vs. Money
**HPF vs. HPF**				
**HPF vs. Money**	0.32 ****			
**Money vs. HPF**	0.31 ****	−0.13		
**Money vs. Money**	0.35 ****	0.52 ****	0.20 **	
**YFAS Symptom Count**	0.01	0.13	−0.08	0.18 *

Note: HPF = Hyper-Palatable Food, YFAS = Yale Food Addiction Scale; $p < 0.0001$, ****, $p < 0.01$, **, $p < 0.05$, *.

Table S2. Pearson Correlations between Single and Cross-commodity Conditions and Large Magnitude Conditions and YFAS Symptom Score.

Conditions	HPF vs. HPF	HPF vs. Money	Money vs. HPF	Money vs. Money
**HPF vs. HPF**				
**HPF vs. Money**	0.18 *			
**Money vs. HPF**	0.39 ****	−0.14		
**Money vs. Money**	0.36 ****	0.56 ****	0.14	
**YFAS Symptom Count**	0.03	0.18 *	−0.19	0.12

Note: HPF = Hyper-Palatable Food, YFAS = Yale Food Addiction Scale; $p < 0.0001$, ****, $p < 0.05$, *.

Table S3. Zero-Inflated Negative Binomial Regression with Large Magnitude Discounting Conditions and Food Addiction Symptomology (N = 285).

Condition	Model (Count/Logit)	IRR/OR (95% CI)	SE	*p* Value
**HPF vs. HPF**	Count	1.04 (0.99–1.11)	0.03	0.207
	Logit	1.04 (0.96–2.35)	0.04	0.346
**Money vs. HPF**	Count	0.95 (0.90–1.00)	0.03	0.059
	Logit	1.06 (0.96–1.15)	0.04	0.174
**HPF vs. Money**	Count	1.05 (0.94–1.13)	0.05	0.316
	Logit	0.90 (0.31–1.02)	0.06	0.071
**Money vs. Money**	Count	1.02 (0.94–1.11)	0.04	0.735
	Logit	0.89 (0.57–1.02)	0.06	0.063

Note: Hunger was included as a covariate in all models. CI = Confidence Interval; HPF = Hyper-Palatable Food; IRR = Incidence Rate Ratio; OR = Odds Ratio; Logit refers to binary portion of the zero-inflated negative binomial model.

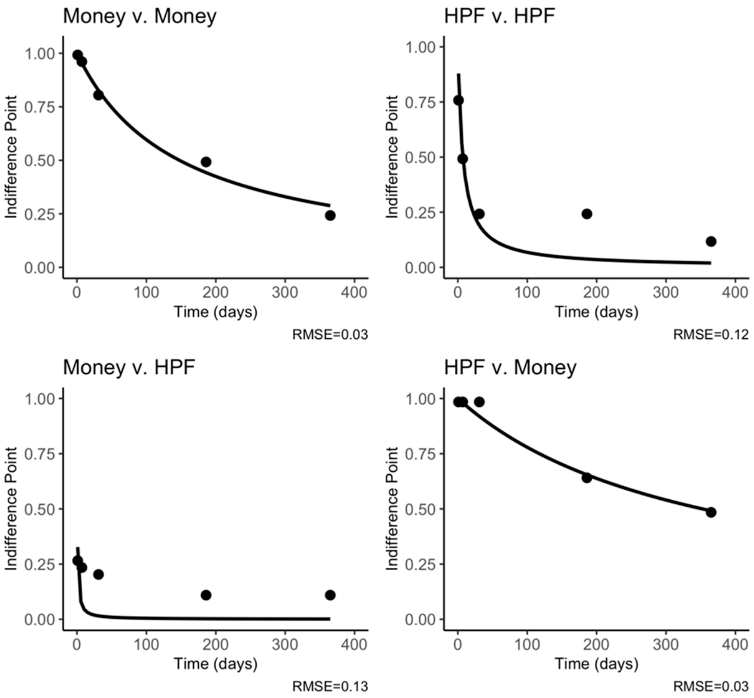

Figure S1. The median indifference point as a function of delay in days for small magnitude conditions using Mazur (1987) hyperbolic model. *Note:* RMSE = Root Mean Squared Error.

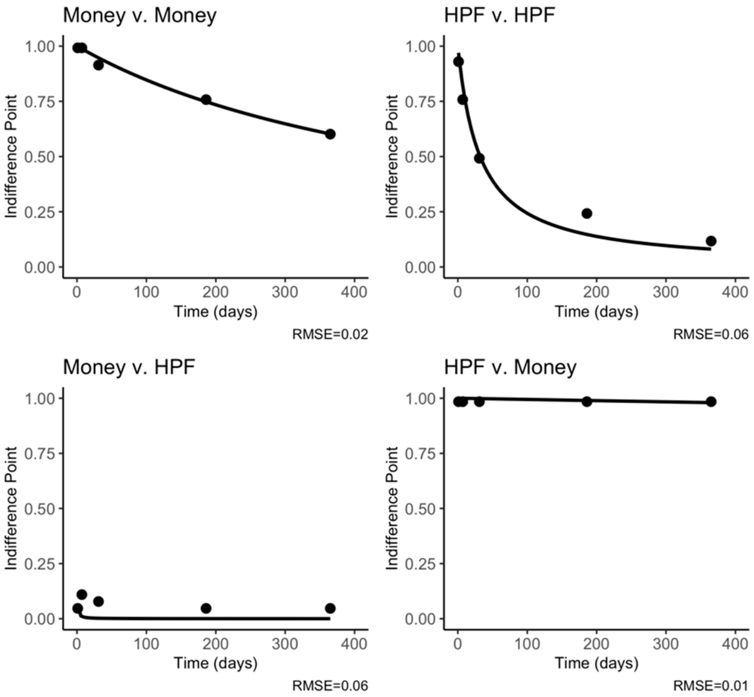

Figure S2: The median indifference point as a function of delay in days for large magnitude conditions using Mazur (1987) hyperbolic model. *Note:* RMSE = Root Mean Squared Error.
